# The heparin-binding domain of VEGF165 directly binds to integrin αvβ3 and VEGFR2/KDR D1: a potential mechanism of negative regulation of VEGF165 signaling by αvβ3

**DOI:** 10.3389/fcell.2024.1347616

**Published:** 2024-05-09

**Authors:** Yoko K. Takada, Jessica Yu, Xiaojin Ye, Chun-Yi Wu, Brunie H. Felding, Masaaki Fujita, Yoshikazu Takada

**Affiliations:** ^1^ The Department of Dermatology, Sacramento, CA, United States; ^2^ The Department of Biochemistry and Molecular Medicine, University of California Davis School of Medicine, Sacramento, CA, United States; ^3^ The Scripps Research Institute, Department of Molecular and Experimental Medicine, La Jolla, CA, United States

**Keywords:** VEGF165, integrin, VEGF receptor, angiogenesis, mutagenesis

## Abstract

VEGF-A is a key cytokine in tumor angiogenesis and a major therapeutic target for cancer. VEGF165 is the predominant isoform of VEGF-A, and it is the most potent angiogenesis stimulant. VEGFR2/KDR domains 2 and 3 (D2D3) bind to the N-terminal domain (NTD, residues 1–110) of VEGF165. Since removal of the heparin-binding domain (HBD, residues 111–165) markedly reduced the mitogenic activity of the growth factor, it has been proposed that the HBD plays a critical role in the mitogenicity of VEGF165. Here, we report that αvβ3 specifically bound to the isolated VEGF165 HBD but not to VEGF165 NTD. Based on docking simulation and mutagenesis, we identified several critical amino acid residues within the VEGF165 HBD required for αvβ3 binding, i.e., Arg123, Arg124, Lys125, Lys140, Arg145, and Arg149. We discovered that VEGF165 HBD binds to the KDR domain 1 (D1) and identified that Arg123 and Arg124 are critical for KDR D1 binding by mutagenesis, indicating that the KDR D1-binding and αvβ3-binding sites overlap in the HBD. Full-length VEGF165 mutant (R123A/R124A/K125A/K140A/R145A/R149A) defective in αvβ3 and KDR D1 binding failed to induce ERK1/2 phosphorylation, integrin β3 phosphorylation, and KDR phosphorylation and did not support proliferation of endothelial cells, although the mutation did not affect the KDR D2D3 interaction with VEGF165. Since β3-knockout mice are known to show enhanced VEGF165 signaling, we propose that the binding of KDR D1 to the VEGF165 HBD and KDR D2D3 binding to the VEGF165 NTD are critically involved in the potent mitogenicity of VEGF165. We propose that binding competition between KDR and αvβ3 to the VEGF165 HBD endows integrin αvβ3 with regulatory properties to act as a negative regulator of VEGF165 signaling.

## 1 Introduction

It has been proposed that growth factor signaling requires integrins for cell responses to growth factor ligation of their cognate cell-surface receptors (Giancotti and Ruoslahti, 1999; Hynes, 2002). The specific mechanisms and the extent of this integrin–growth factor crosstalk are still not fully established. Integrins are cell-surface heterodimers that act as receptors for extracellular matrix ligands, cell-surface ligands (e.g., ICAM-1 and VCAM-1), and soluble ligands that include growth factors (Hynes, 2002; Takada et al., 2007). The finding that integrin αvβ3 antagonists inhibit fibroblast growth factor-2 (basic FGF and FGF2) signaling (Brooks et al., 1994) suggested that αvβ3 is involved in growth factor signaling through a crosstalk mechanism (Desgrosellier and Cheresh, 2010; Kim et al., 2011; Odenthal et al., 2016). We previously reported that FGF1 and FGF2 directly bind to integrin αvβ3, leading to the formation of an integrin–FGF–FGFR ternary complex that is required for FGF signaling functions (the ternary complex model) (Mori et al., 2008; Yamaji et al., 2010; Mori et al., 2013; Mori et al., 2017). We showed that this model can be applied to other growth factors as well, including IGF1, IGF2, neuregulin-1, fractalkine, and CD40L (Saegusa et al., 2009; Ieguchi et al., 2010; Fujita et al., 2012; Cedano Prieto et al., 2017; Takada et al., 2019). Thus, direct binding of integrin αvβ3 to the growth factors is required for their signaling functions. Notably, we showed that growth factor mutants defective in integrin binding are also defective in signaling functions and can act as antagonists of the signaling process, even though the growth factor mutants still bind to their cognate receptors (Mori et al., 2008; Yamaji et al., 2010; Fujita et al., 2012; Mori et al., 2013; Cedano Prieto et al., 2017; Mori et al., 2017; Takada et al., 2019).

Vascular endothelial growth factor (VEGF-A) is a key cytokine in physiological and pathological angiogenesis and a major therapeutic target. It has been proposed that VEGF-A signaling requires integrin αvβ3, but it is unclear whether the ternary complex model can be applied since VEGF-A binding to αvβ3 has not been documented. Among the six main isoforms of VEGF-A, VEGF165 (the 165-amino-acid protein) is the predominant gene product in human tissues and the most potent angiogenesis stimulant (Ferrara et al., 1996; Ferrara, 2004). VEGF165 forms homodimers of two anti-parallel monomers that interact via their N-terminal domains, which harbor the cognate receptor-binding site. VEGF165 exerts mitogenicity by binding to the VEGFR1 (FLT1) and VEGFR2 (KDR) receptor tyrosine kinases (Waltenberger et al., 1994). KDR, a 230-kDa glycoprotein, has a lower affinity for VEGF165 (KD = 0.75–2 x 10^−10^ M) than VEGFR1 (KD 1–2 × 10^−11^ M). Yet, KDR is the primary mediator of VEGF165 signaling (Gille et al., 2001). Within its N-terminal region, KDR has seven IgG-like extracellular domains, of which domains 2 (D2) and 3 (D3) interact with the N-terminal domain of VEGF165 with high affinities (Gille et al., 2001). The clinically used anti-angiogenic monoclonal antibody Avastin (bevacizumab) binds to the N-terminal domain of VEGF165 (residues 1–110) and inhibits VEGF165 binding to KDR. The C-terminal domain of VEGF165 encompasses a heparin-binding domain (HBD, residues 111–165) and a neuropilin-binding site. A plasmin-cleavage site located between the ligand’s N-terminal and heparin-binding domains enables proteolytic removal of the HBD, which markedly reduces the mitogenicity of VEGF165 in endothelial cells (Keyt et al., 1996). This suggests that KDR binding to the N-terminal VEGF165 domain may not be sufficient to exert a cell growth response when the HBD is missing. The reduced mitogenic activity of a mutant VEGF165 homodimer that lacks the HBD is like the mitogenicity observed for VEGF121, a natural splice variant that lacks exons 6a and 7, which encode most of the HBD. Although evidence suggests that the HBD plays a role in VEGF165-dependent processes, including angiogenesis, it is unclear how the HBD contributes to VEGF165-mediated cell responses. It has been reported that RGD-disintegrin from *Bothrops alternatus* venom binds to αvβ3 integrin with nanomolar affinity and blocks cell adhesion to the extracellular matrix (Danilucci et al., 2019). This disintegrin directly interferes with αvβ3/KDR crosstalk and its downstream pathways (Danilucci et al., 2019). In addition, it has been reported that angiogenesis induced by VEGF165 can be inhibited by RGD-containing disintegrin from ADAM15 (Kim et al., 2023), which specifically binds to integrin αvβ3 (Zhang et al., 1998). However, the specifics of the role of αvβ3 in VEGF165 or KDR signaling are unclear. It has been reported that β3 knockout in a mouse model enhances angiogenesis and tumorigenesis (Reynolds et al., 2002) and accelerates wound healing (Reynolds et al., 2005). Thus, it has been proposed that integrin αvβ3 negatively regulates VEGF signaling.

In the present study, we first showed that αvβ3 specifically bound to the isolated HBD of VEGF165 but not to the isolated NTD of VEGF165. To understand the binding mechanism, we applied docking simulations and mutagenesis and found that integrin αvβ3 binding to VEGF165 critically involves amino acid residues within the VEGF165 HBD, namely, Arg123, Arg124, Lys125, Lys140, Arg145, and Arg149. Unexpectedly, we discovered that the VEGF165 HBD binds to KDR domain 1 (D1), and the D1-binding site in the HBD overlaps with that of αvβ3. A full-length VEGF165 expressing the combined HBD mutations (R123A/R124A/K125A/K140A/R145A/R149A) was defective in integrin binding and KDR D1 binding. Importantly, the full-length VEGF165 mutant defective in integrin and KDR D1 binding failed to induce ERK1/2 phosphorylation, integrin β3 phosphorylation, and KDR phosphorylation and did not support the proliferation of endothelial cells, although the mutation did not affect KDR D2D3 binding to VEGF165. Since β3-knockout mice showed enhanced VEGF signaling, we propose that VEGF165 binds to KDR D2D3 and KDR D1 on the cell surface and that this process is critically involved in the potent mitogenicity of VEGF165. It is likely that integrin αvβ3 competes with KDR D1 for binding to the VEGF165 HBD and acts as a negative regulator of VEGF165 signaling.

## 2 Materials and methods

Recombinant soluble αvβ3 was synthesized in Chinese hamster ovary (CHO) K1 cells using the soluble αv and β3 expression constructs, and the recombinant proteins were purified by nickel-nitrilotriacetic acid (Ni-NTA) affinity chromatography, as described by Takagi et al. (2001). Anti-phospho-integrin β3 (Tyr747) and rabbit polyclonal anti integrin β3 (pY773) were purchased from Invitrogen. HRP-conjugated anti-His tag antibody and HRP-conjugated anti-GST antibody were purchased from QIAGEN (Valencia, CA, United States). Mab 7E3 (anti-human integrin β3) hybridoma was obtained from ATCC. Anti-phospho-KDR (Tyr1175), rabbit mAb (19A10), anti-ERK1/2 (p44/42), and anti-phospho p44/42 (Thr202/Tyr204) were obtained from Cell Signaling Technologies (Danvers, MA, United States). Rabbit polyclonal anti-integrin β3 was obtained from Chemi-Con/Sigma-Aldrich. KDR/Fc chimera was obtained from R&D systems (Minneapolis, MN, United States).

### 2.1 Plasmid construction and protein purification

The cDNA fragment of the VEGF165 N-terminal domain (NTD) (APMAEGGGQNHHEVVKFMDVYQRSYCHPIETLVDIFQEYPDEIEYIFKPSCVPLMRCGGCCNDEGLECVPTEESNITMQIMRIKPHQGQHIGEMSFLQHNKCECRPKKDRARQEN) was amplified by PCR using primers 5′-GGG​GAT​CCG​CAC​CCA​TGG​CAG​AAG​GAG​G-3′ and 5′-GGA​ATT​CTC​AAT​CtT​TCT​TTG​GTC​TGC​ATT​C-3′ with human VEGF165 cDNA (Open Biosystems, Lafayette, CO, United States) as a template, and it was sub-cloned into the BamHI/EcoRI site of the PET28a expression vector. The VEGF165 heparin-binding domain (HBD) (PCGPCSERRKHLFVQDPQTCKCSCKNTDSRCKARQLELNERTCRCDKPRR) of VEGF165 was amplified by PCR using primers 5′-CGG​GAT​CCC​CCT​GTG​GGC​CTT​GCT​CAG​AG-3′ and 5′-CGG​AAT​TCT​CAC​CGC​CTC​GGC​TTG​TCA​CAT​C-3′ with human VEGF165 cDNA (Open Biosystems, Lafayette, CO, United States) as a template, and it was sub-cloned into the BamHI/EcoRI site of the PET28a expression vector. The protein was synthesized in BL21 induced by IPTG as an insoluble protein and solubilized in 8 M urea, purified by Ni-NTA affinity chromatography, and refolded as described by Saegusa et al. (2009).

The cDNA fragment of KDR D1 (NTTLQITCRGQRDLDWLWPNNQSGSEQRVEVTECSDGLFCKTLTIPKVIGNDTGAYKCFYRETDL) was amplified by PCR using primers 5′-CGG​GAT​CCG​ACA​TAC​TTA​CAA​TTA​AGG​C-3′.

5′-CGG​AAT​TCT​CAA​GAT​CTG​TAA​TCT​TGA​ACA​TAG-AC-3′ with full-length KDR cDNA and sub-cloned into the BamHI/EcoRI site of PET28a vector, and protein was synthesized in BL21 induced by IPTG as an insoluble protein. The insoluble protein was solubilized in 8 M urea, purified by Ni-NTA affinity chromatography, and refolded as described by Saegusa et al. (2009). The cDNA fragment was sub-cloned into the BamHI/EcoRI site of pGEX2T vector, and protein was expressed in BL21 and purified by Glutathione–Sepharose affinity chromatography.

Site-directed mutagenesis was carried out as described by Saegusa et al. (2009). The presence of the mutation was verified by DNA sequencing.

### 2.2 Surface plasmon resonance study of the αvβ3-HBD interaction

Soluble αvβ3 (His-tagged) was immobilized on the CM5 sensor chip using a standard amine coupling procedure in HBS-P buffer (0.01 M HEPES, pH 7.4, 0.15 M NaCl, and 0.0005% of surfactant P20) with 1 mM of Mn^2+^. The HBD was injected at 50 μL/min for 1.8 min. HBS-P buffer with 1 mM of Mn^2+^ was then injected at 50 μL/min for 3 min to allow bound VEGF to dissociate from αvβ3.

### 2.3 Surface plasmon resonance study of the KDR D1-HBD interaction

KDR D1 (His-tagged) was immobilized on the CM5 sensor chip using a standard amine coupling procedure in HBS-EP buffer (0.01 M HEPES, pH 7.4, 0.15 M NaCl, 3 mM EDTA, and 0.0005% of surfactant P20). The HBD was injected at 50 μL/min for 1.8 min. The HBS-EP buffer was then injected at 50 μL/min for 3 min to allow the bound VEGFs to dissociate from the KDR D1.

### 2.4 ELISA-type αvβ3 binding assay

Wells of 96-well microtiter plates were coated with PBS-diluted protein, as specified in the experiments, and incubated at 37°C for 1 h. The wells were then blocked with 0.1%BSA/PBS boiled at 80°C for 20 min to reduce background and then cooled to room temperature before application of 300 μL/well. After a 30-min incubation at room temperature, soluble αvβ3 (1 μg/mL) in HEPES-Tyrode buffer + 1 mM MnCl_2_ (incubation buffer) was added and plates were further incubated at room temperature for 1 h. Unbound αvβ3 was removed by washing with incubation buffer, and bound αvβ3 was quantified using anti-β3 mAb (AV10), followed by HRP-conjugated anti-mouse IgG and peroxidase substrate 3,3′,5,5′-tetramethylbenzidine (TMB) solution, 100 μL/well. The reaction was stopped by the application of 2N HCl, 50 μL/well, and measurements were taken at OD = 450 nm with a plate reader.

### 2.5 ELISA-type KDR D1 binding assay

Wells of 96-well microtiter plates were coated with HBD and incubated at 37°C for 1 hour. The wells were then blocked with 0.1% BSA/PBS boiled at 80°C for 20 min and then cooled before application of 300 μL/well. After 1 h of blocking, wells were incubated with KDR D1 fused to GST for 1 h at room temperature. After washing with 0.05% Tween 20/PBS, an anti-GST conjugated with HRP was applied for 1 hour at room temperature. Three washes were performed with 0.05% Tween-20/PBS before detection with the TMB solution, 100 μL/well. The reaction was stopped by the application of 2N HCl, 50 μL/well, and measurements were taken at OD = 450 nm with a plate reader.

### 2.6 Docking simulation

Docking simulation of the interaction between the VEGF165 HBD (2VGH.pdb) and integrin αvβ3 was performed using AutoDock3, as described by Saegusa et al. (2009). In the present study, we used the headpiece (residues 1–438 of αv and residues 55–432 of β3) of αvβ3 (open-headpiece form, 1L5G.pdb). Cations were not present in αvβ3 during docking simulation (Mori et al., 2008; Saegusa et al., 2008; Fujita et al., 2015). We also performed a docking simulation without removing cations (Supplementary Figure S1).

### 2.7 SDS-PAGE analysis of wild-type and mutant VEGF165

In mutant VEGF165, amino acids R123, R124, K125, K140, R145, and R149 within the HBD were identified as required for integrin αvβ3 binding based on changing these residues to Ala (R123A/R124A/K125A/K140A/R145A/R149A) either in sterically relevant clusters or in combination. Molecular size values are shown in kDa.

### 2.8 Pull-down of VEGF165 by KDR

His-tagged KDR was immobilized on Ni-NTR beads and incubated with full-length VEGF165 wild-type (WT) vs. VEGF165 mutant (mut) protein in the binding buffer (10 mM Tris, 0.15 M NaCl, pH 7.5) for 2 h at 4°C before elution and analysis of the retained protein by SDS-PAGE. Both wild-type and mutant VEGF165 bind to KDR.

### 2.9 ERK1/2 activation in HUVECs by VEGF165

Human umbilical vein endothelial cells (HUVECs) were starved for 4 h in M200 basal medium without low serum growth supplement and then treated with VEGF165 wild-type or mutant protein (10 ng/mL) for 10 min before lysis and Western blot analysis. Cells were lysed in lysis buffer 20 mM HEPES (pH 7.4), 100 mM NaCl, 10% glycerol, 1% Nonidet P-40, 1 mM MgCl_2_, 1 mM PMSF, 20 mM NaF, 1 mM Na_3_VO_4_, and protease inhibitor mixture (Sigma-Aldrich). Cell lysates were analyzed by Western blotting using specific antibodies directed to ERK1/2 (p44/42) or phospho ERK1/2 p44/42 (Thr202/Tyr204). Bound IgG was detected using the HRP-conjugated second antibody and SuperSignal West Pico (Thermo Fisher Scientific). Images were evaluated using a Fuji LAS 4000 mini luminescent image analyzer and Multi Gauge v.3.0 software (Fujifilm, Tokyo, Japan).

### 2.10 Integrin αvβ3 phosphorylation in HUVECs in response to VEGF165

Starved HUVECs, as detailed above, were treated with VEGF165 wild-type vs. mutant (10 ng/mL) for 10 min before lysis and Western blot analysis of β3 integrin subunit phosphorylation using anti-phospho β3 antibodies.

### 2.11 KDR Y1175 phosphorylation in HUVECs in response to VEGF165

Starved HUVECs were treated for Western blot analysis**.** We stimulated the starved endothelial cells with WT and/or mutant VEGF165 for 10–20 min. We solubilized cells in lysis buffer (20 mM HEPES (pH 7.4), 100 mM NaCl, 10% glycerol, 1% Nonidet P-40, 1 mM MgCl_2_, 1 mM PMSF, 20 mM NaF, 1 mM Na_3_VO_4_, and protease inhibitor mixture (Sigma-Aldrich)). We analyzed cell lysates by Western blotting using specific antibodies. Bound IgG was detected using HRP-conjugated second antibody and SuperSignal West Pico (Thermo Fisher Scientific). We analyzed the images using a Fuji LAS 4000 mini luminescence image analyzer and Multi Gauge v.3.0 software (Fujifilm, Tokyo, Japan).

### 2.12 Statistical analysis

Results are reported as the mean ± standard error of the mean and calculations performed using Prism 10, GraphPad. Where indicated, statistical analysis was performed using ANOVA.

## 3 Results

### 3.1 Direct binding of integrin αvβ3 to the VEGF165 heparin-binding domain

**FIGURE 1:**
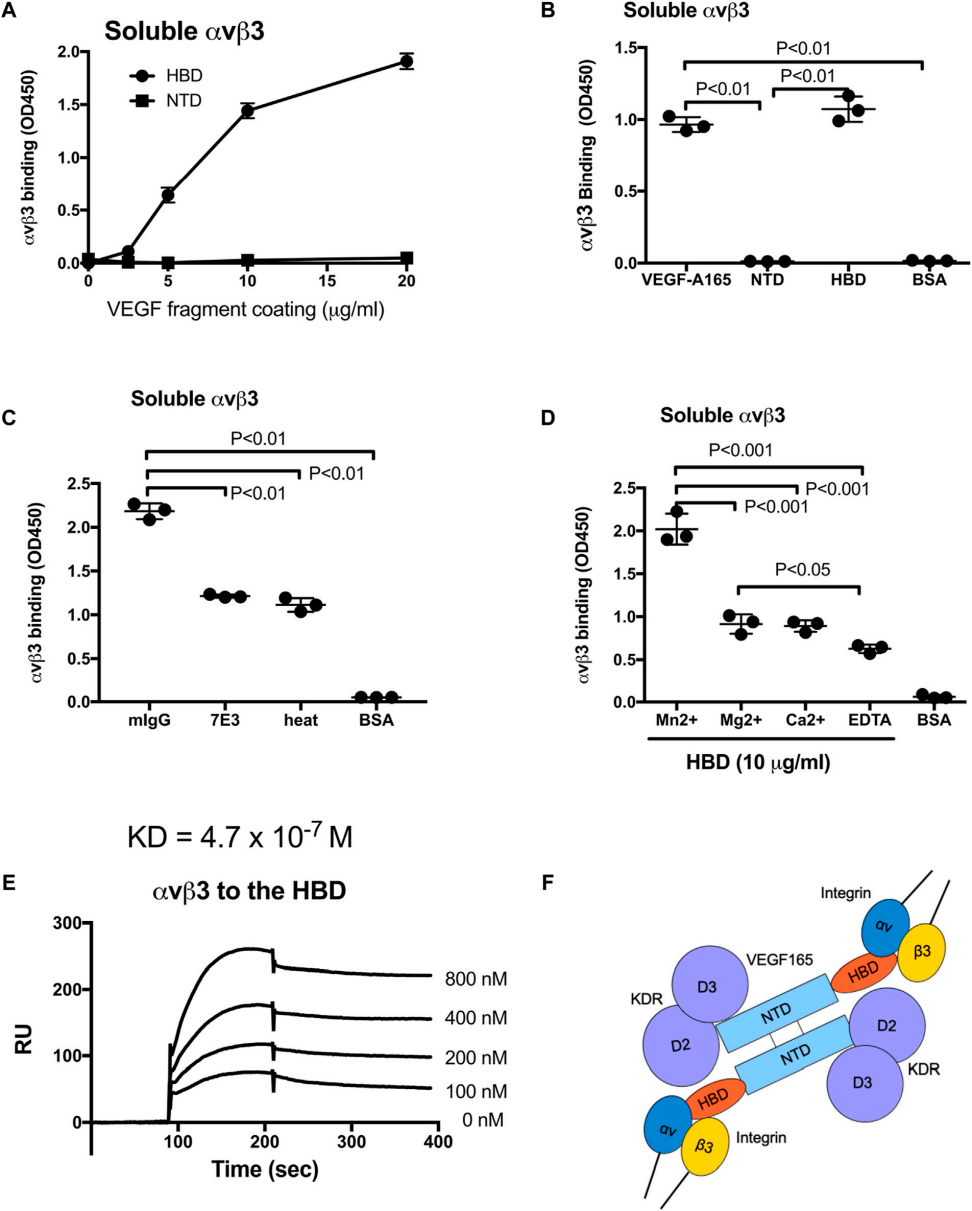
Integrin αvβ3 binds to the heparin-binding domain (HBD, residues 111–165) of VEGF165 but not to the N-terminal domain (NTD, residues 1–110). **(A,B)** Interaction of soluble αvβ3 with immobilized VEGF165 HBD vs. NTD. Microtiter 96-wells were coated with increasing concentrations of VEGF165 or its fragments and incubated with soluble αvβ3 after blocking non-specific protein-binding sites with BSA. **(B)** Control wells were coated with BSA. αvβ3-binding was detected with non-function blocking anti-β3 mAb AV10. **(C)** Specificity of αvβ3 binding to the VEGF165-HBD documented based on the inhibition of αvβ3 binding by function-blocking anti-β3 antibody, mAb 7E3, or heat treatment but not by control mouse IgG (mIgG). Control wells were coated with BSA. **(D)** Cation dependence of αvβ3 binding to the VEGF165-HBD. Binding of soluble αvβ3 to HBD protein (10 μg/mL) immobilized on microtiter plates and blocked with BSA in the absence of divalent cations (EDTA) or presence of Mn^2+^, Ca^2+^, or Mg^2+^ (1 mM). Control wells were coated with BSA. **(E)** Surface plasmon resonance analysis of the αvβ3–HBD interaction. αvβ3 protein was immobilized to a sensor chip, and binding of the solubilized mobile HBD protein was measured as the analyte at increasing concentrations. Where applicable, data are shown as means +/− SD of triplicate experiments. **(F)** Model of αvβ3 binding to the C-terminal HBD within a VEGF165 homodimer. The NTD is known to bind to KDR domains 2 and 3 (D2D3). The present study showed that αvβ3 binds to the HBD but not to the NTD. We predict that αvβ3, VEGF165, and KDR generate the ternary complex on the cell surface.

It has been proposed that extracellular matrix-bound VEGF165 interacts with integrin αvβ3 expressed by endothelial cells, and evidence indicates that VEGF121, which lacks the HBD, does not have this property (Hutchings et al., 2003). This implies that the HBD of VEGF165 likely contains a binding site for αvβ3. However, the specifics of an HBD–αvβ3 interaction and the possible cellular consequences are unclear. In this study, we directly addressed the question of whether integrin αvβ3 recognizes and binds the VEGF165 HBD, and if so, whether this interaction can initiate αvβ3 activation at the molecular level and trigger ERK1/2 activation in endothelial cells as a first indication of endothelial cell responses known to be involved in VEGF165-stimulated functions. We found that soluble αvβ3 bound to immobilized VEGF165-HBD protein in a dose-dependent manner but not to the VEGF165 N-terminal domain (NTD) in an ELISA-type binding assay (Figures 1A,B). This binding was suppressed by heat treatment of the HBD, suggesting that the interaction requires native folding of this domain. αvβ3-HBD binding was inhibited by anti-integrin β3 function blocking mAb 7E3 (Figure 1C), whose epitope has been mapped to the classical ligand-binding site within the β3 integrin subunit (Puzon-McLaughlin et al., 2000; Artoni et al., 2004). The inhibitory effects of integrin antagonists suggest that the VEGF165 HBD binding to αvβ3 is specific. Furthermore, we found that binding of soluble αvβ3 to the VEGF165 HBD is cation-dependent (Mn^2+^>Mg^2+^ = Ca^2+^>EDTA) (Figure 1D) in a manner consistent with the reported αvβ3 ligand binding. In agreement with this observation, soluble αvβ3 is known to require 1 mM Mn^2+^ for full activation, as we previously documented (Mori et al., 2008). Moreover, we previously showed that several cytokines directly bind to soluble integrins, prominently including αvβ3 and its interaction with FGF1 and IGF1 (Mori et al., 2008; Saegusa et al., 2009). Thus, the present findings on the αvβ3-VEGF165 HBD interaction support the concept of a key role of integrin αvβ3 interaction with growth factors that have so far been thought to exert their biological properties primarily, if not exclusively, via their known cognate receptors (de Laat et al., 1999). Supporting this concept, our results documenting the cation requirement of VEGF165 HBD–αvβ3 binding and its inhibition by β3 function blocking mab 7E3, as well as the abrogation of this molecular interaction by heat denaturation of the HBD, measured by ELISA-type binding assays to investigate the properties of the purified binding partners reported here, are in line with our previous findings that the FGF1 and IGF1 interaction with integrin αvβ3 can modulate cell signaling responses to these growth factors. Using surface plasmon resonance analysis as an orthogonal approach to study the HBD-αvβ3 interaction and determine the binding affinity, K_D_ was calculated as 4.7 × 10^−7^ M (Figure 1E). Collectively, our results suggest that the VEGF165 HBD is a specific ligand for integrin αvβ3 (Figure 1F). These findings predict that VEGF165 binds to αvβ3 through the HBD and to KDR via the N-terminal domains (NTD), likely resulting in an integrin–VEGF165–KDR ternary complex (Figure 1F).

Although αvβ3 binds to extracellular matrix (ECM) ligands such as fibronectin and vitronectin, it is not straightforward to compare the αvβ3–VEGF165 HBD interaction to αvβ3–ECM interactions since αvβ3–ECM interactivity has mostly been measured based on cell adhesion assays. Cell adhesion assays measure cell interactions with their substrates mediated by multiple integrins and multiple ECM ligands, potentially including additional non-matrix molecules such as growth factors that bind to ECM, all of which may contribute to the interaction and cell adhesion process (Janus-Bell and Mangin, 2023; Sleeboom et al., 2024)*.* Our present study, by design, directly addresses the molecular interaction between αvβ3 as a single integrin and the HBD of VEGF165 as a specific region within a single ligand whose effects on cell behavior and vascular responses have so far been attributed to VEGF165 recognition by its cognate receptor KDR. The present findings demonstrate that the VEGF165 HBD specifically binds to integrin αvβ3.

### 3.2 Mapping amino acid residues within the VEGF165 HBD for integrin binding

**FIGURE 2:**
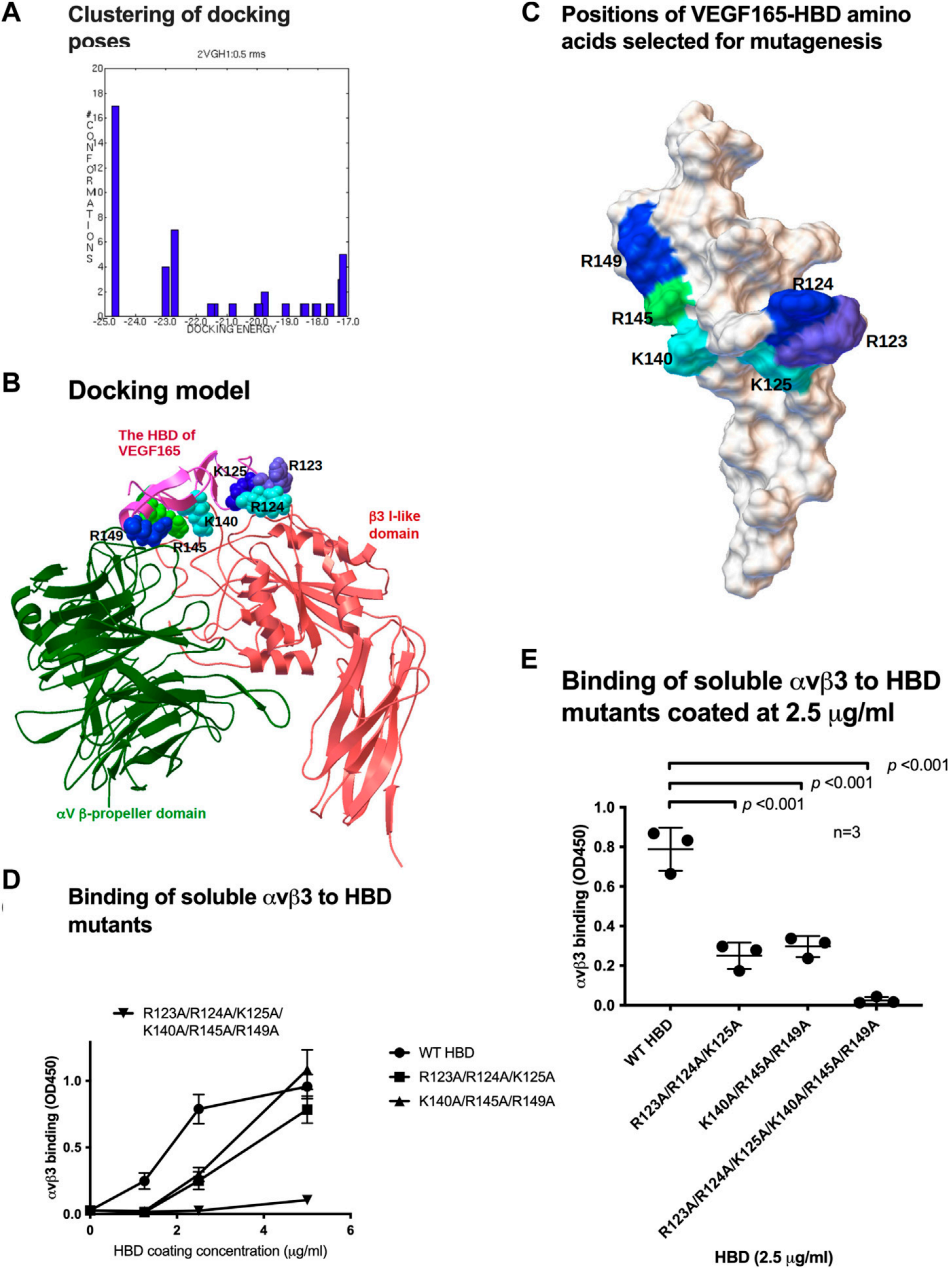
Mapping the integrin-binding sites within the heparin-binding domain (HBD) of VEGF165. **(A)** Clustering of docking poses. Docking simulation of the interaction between the HBD (2VGH.pdb) and αvβ3 (1 L5G.pdb) was performed using AutoDock 3. Docking poses (total 50) were clustered (<0.5 RMS). Majority of the poses (17) were clustered in the first cluster (docking energy −24.6 kcal/mol). They are most likely poses in which HBD binds to integrin αvβ3. **(B)** Docking model of the interaction between integrin αvβ3 and the HBD based on docking simulation. HBD amino acid residues predicted to contribute to αvβ3 binding are Arg-123, Arg-124, Lys-125, Lys-140, Arg-145, and Arg-149. **(C)** Position of HBD amino acids within the predicted integrin-binding interface was selected for mutagenesis and changed to Ala in combinations. **(D)** Binding of soluble αvβ3 to VEGF165-HBD mutants coated onto microtiter wells at increasing concentrations. Non-specific binding sites were blocked with BSA. **(E)** Binding of soluble αvβ3 to VEGF165-HBD mutants coated at a near-saturation concentration (2.5 μg/mL) and identified for the wild-type (WT) HBD protein revealed that all HBD amino acids predicted to contribute to αvβ3 binding are required for the VEGF165-integrin αvβ3 interaction. Where applicable, data are shown as means +/− SD of triplicate experiments.

To predict which amino acid residues are involved in αvβ3 binding, we performed docking simulations between the HBD (2VGH.pdb) and αvβ3 (1L5G.pdb, with open headpiece) using AutoDock 3 (Figure 2A). We performed 50 cycles of docking simulation, and all docking poses were clustered (<0.5 root-mean-square deviation (RMSD)). Seventeen (34%) of the docking poses were in the first cluster. The pose in the first cluster with the lowest docking energy (−24.6 kcal/mol) was selected for further analysis. The simulation predicted that the HBD binds to αvβ3 at high affinity. Amino acid residues predicted to be involved in the HBD–αvβ3 interaction are Arg123, Arg124, Lys125, Lys140, Arg145, and Arg149 (Table 1). We selected these Arg and Lys residues of the VEGF165 HBD at the predicted binding interface for mutagenesis (Figure 2B). Binding of soluble αvβ3 to immobilized HBD wild-type vs. mutant protein was measured by ELISA-type binding assays. The Arg123/Arg124/Lys125 to Ala (R123A/R124A/K125A) mutation and the Lys140/Arg145/Arg149 to Ala mutation (K140A/R145A/R149A) partially suppressed the binding of αvβ3 to wild-type HBD (Figure 2C). The combined R123A/R124A/K125A/K140A/R145A/R149A mutation nearly completely blocked integrin binding (Figures 2D, E). These results indicate that these amino acid residues are critical for VEGF165 HBD binding to αvβ3, and they are consistent with the docking model.

**TABLE 1:** Amino acid residues predicted to be involved in HBD binding to integrin αvβ3. Amino acid residues within 0.6 nm between the HBD and αvβ3 were selected using Swiss-PDBViewer (version 4.1) (Swiss Institute of Bioinformatics, Basel, Switzerland).

HBD	αv	β3
Glu122, Arg123, Arg124, Lys 125, His126, Leu127, Phe128, Val129, Gln130, Asp131, Pro132, Gln133, Lys140, Ser144, Arg145, Cys146, Ala148, Arg149, Gln150, Leu151, and Cys160	Ala149, Asp150, Gly151, Phe177, Tyr178, Gln180, Arg211, Thr212, Ala213, Gln214, Ala215, and Asp218	Tyr122, Ser123, Met124, Lys125, Asp126, Asp127, Met180, Lys181, Thr182, Thr183, Arg214, Asn215, Asp251, Thr334, Met335, Asp336, and Ser337

The integrin αvβ3 structure (1L5G.pdb) contains eight Mn^2+^ cations. In our initial docking simulations, all these cations were removed from the integrin structure (Figure 2A). Since cations are critical for integrin binding, it is possible that an integrin structure without cations may not reflect ligand binding in biological conditions. We thus performed docking simulation using the αvβ3 integrin in which cations were not removed. We obtained an identical docking model (−22.5 kcal/mol) in the first cluster (Supplementary Figure S1). This indicates that the presence or absence of cations did not affect the prediction by docking simulation of the integrin–HBD interaction.

### 3.3 Direct binding of the VEGF165 HBD to the first IgG-like domain of KDR (D1)

**FIGURE 3:**
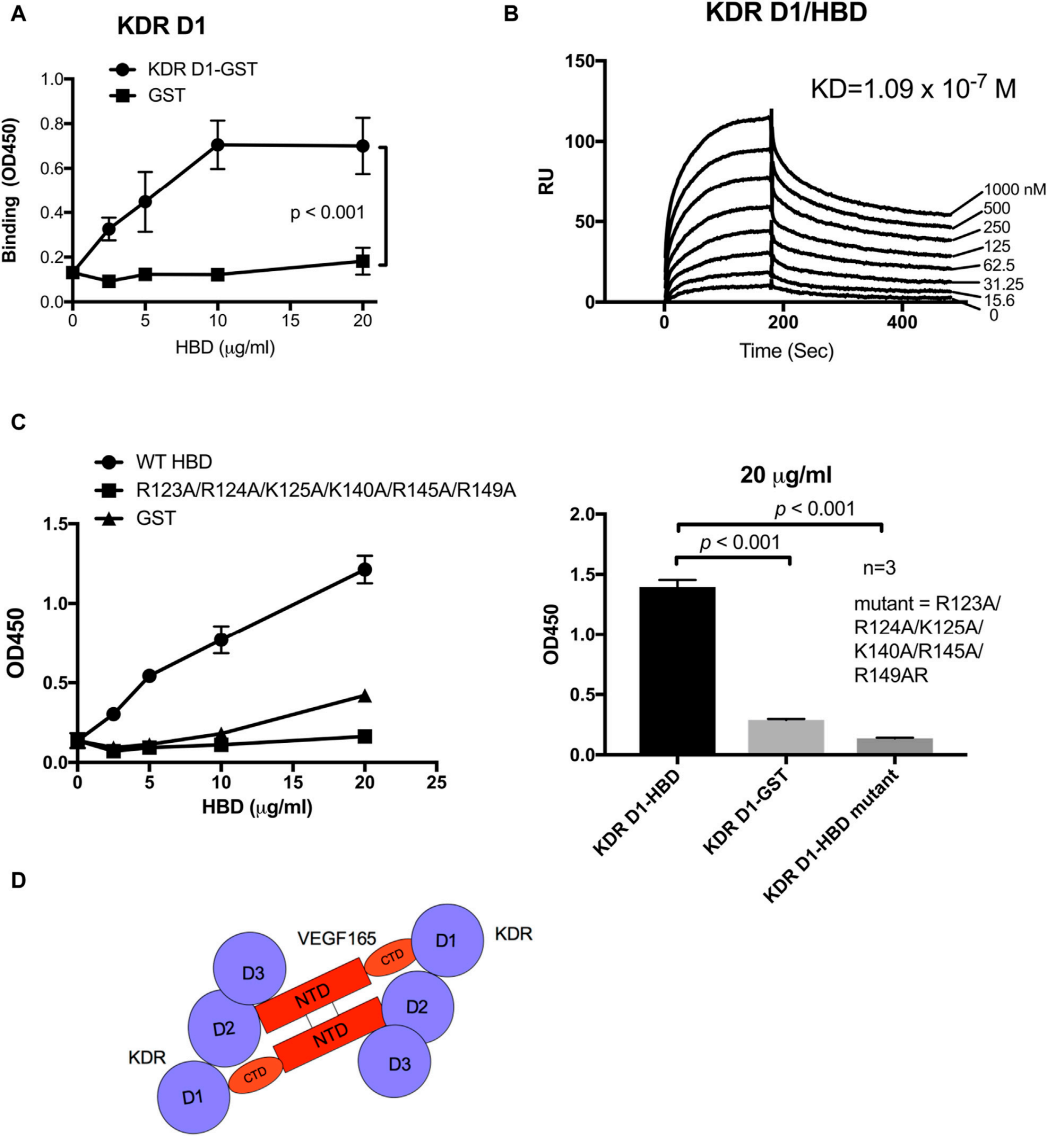
KDR domain 1 (D1) binds to the HBD (residues 111–165) of VEGF165. **(A)** Binding of KDR D1 to HBD. The wells of 96-well microtiter plates were coated with HBD, and remaining protein-binding sites were blocked with BSA. Wells were incubated with soluble KDR fragments (GST-tagged) (100 μg/mL), and bound KDR D1 was measured using HRP-conjugated anti-GST IgG. **(B)** Binding dynamics measured by surface plasmon resonance (SPR) assay of the KDR D1-HBD interaction. KDR D1 was immobilized to a sensor chip, and HBD was dissolved in the solution phase as analytes. **(C)** HBD mutant is defective in binding to KDR D1. The binding of HBD WT and mutant to KDR D1 was measured as described in **(A)**. **(D)** Model of the KDR–VEGF165 interaction. KDR D1 binds to the HBD (or C-terminal domain, CTD), and KDR D2D3 binds to the N-terminal domain.

It has been proposed that integrin αvβ3 negatively regulates VEGF165 signaling since β3 KO mice had elevated levels of angiogenesis and tumorigenesis (Reynolds et al., 2002; Reynolds et al., 2005). Considering these results from β3 KO mouse models, the here proposed ternary complex between αvβ3, VEGF165, and KDR D2D3 may not be required for maximal mitogenicity of VEGF165. Instead, the presence of αvβ3 bound to the VEGF165 HBD might rather act as a negative modulator of VEGF165–KDR binding-induced mitogenic signaling. We, therefore, hypothesized that another protein might bind to the VEGF165 HBD and potentially sterically compete with αvβ3 to regulate the intensity of VEGF165-KDR induced mitogenicity. Since removal of the VEGF165 HBD markedly reduced KDR binding to VEGF165, as previously shown (Keyt et al., 1996) and consolidated in our binding experiments, we hypothesized that the VEGF165 HBD might also be recognized by KDR itself but involves a KDR domain distinct from its D2D3, which engage the VEGF165 NTD. The VEGF165 HBD is strongly positively charged (pI = 11). We, therefore, anticipated that KDR D1 might interact with the VEGF165 HBD as the KDR D1 domain is strongly negatively charged. To test this hypothesis, we performed binding experiments using orthogonal approaches. Applying the ELISA-type binding assay, we demonstrated that the VEGF165 HBD bound to KDR D1 (Figure 3A). This interaction was confirmed by surface plasmon resonance (SPR), which further added information on the binding dynamics (Figure 3B). Thus, we conclude that the VEGF165 HBD interacts with the KDR D1 region. These results indicate that KDR D1 can interact with the VEGF165 HBD and thus ligate VEGF165 via a previously unrecognized binding site.

### 3.4 Binding sites within the VEGF165 HBD for αvβ3 and KDR D1 overlap

**FIGURE 4:**
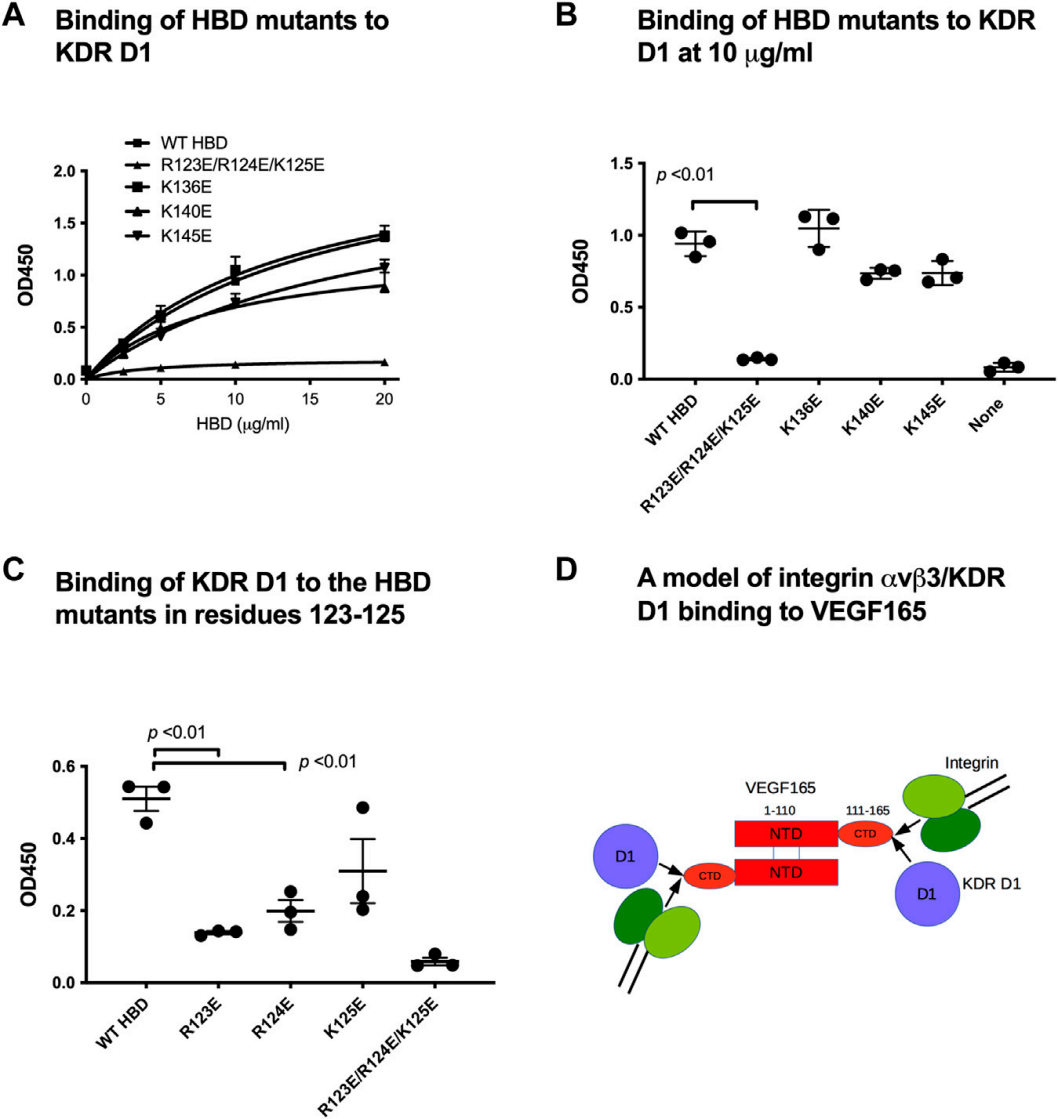
Mapping KDR D1-binding sites in HBD. We hypothesized that basic amino acid residues on the surface of HBD play a role in negatively charged KDR D1. Thus, we mutated lysine (K) and arginine (R) on HBD to glutamic acid (E). Wells of 96-well microtiter plates were coated with His-tagged HBD and incubated with GST-KDR-D1 (100 μg/mL); bound GST were measured using the HRP-conjugated anti-GST antibody. **(A)** Dose–response curve of KDR D1 binding. **(B)** Binding of HBD mutants (at 10 μg/mL). The R122E/R123E/R124E mutant was very defective in KDR D1 binding. **(C)** Individual R123E and R124E mutants are defective in KDR D1 binding. **(D)** Model of integrin and KDR D1 binding to VEGF165. Our data suggest that KDR D1 and αvβ3-binding sites overlap in the HBD (or C-terminal domain, CTD). We predict that KDR D1 and αvβ3 compete for binding to the HBD since their binding affinity is comparable.

Since our results identified a novel, potentially regulatory mechanism through which integrin αvβ3 binding to the VEGF165 HBD impacts cell signaling induced by VEGF165 binding to KDR, we explored the effect of VEGF165 HBD mutations on the newly discovered KDR D1 interaction. We studied the effect of mutations on the binding of KDR-D1 to the VEGF165 HBD. We were not able to use docking simulation between the HBD and the KDR D1 since KDR D1 is not folded and the 3D structure of KDR D1 is not available. We found that the combined mutations within the VEGF165 HBD blocked KDR D1 binding to the HBD of this ligand (Figure 3C). As mutations of these amino acids within the VEGF165 HBD also abrogated the integrin αvβ3 interaction with this ligand (Figure 3C), these findings indicate that the interaction sites of αvβ3 and KDR D1 within the VEGF165 HBD overlap (Figure 3D). To further locate the KDR D1 binding site within the VEGF165 HBD, we introduced point mutations of positively charged amino acid residues to Glu (charge reversal mutations) within the HBD to disturb the proposed charge-supported interaction between the VEGF165 HBD and KDR D1. The R123E/R124E/K125E mutation strongly suppressed the binding of HBD to KDR D1 (Figures 4A and B), and mutations of individual residues, R123E and R124E, but not K125E (Figure 4C), suppressed the binding. These results suggest that Arg123 and Arg124 are critical for KDR D1 binding and that αvβ3- and KDR D1-binding sites overlap in HBD (Table 1). Therefore, it is probable that αvβ3 and KDR D1 compete for binding to the HBD (Figure 4D) and that αvβ3 can interfere with KDR D1 binding to the VEGF165 HBD and, thereby, blunt VEGF165 signaling induced by the KDR–VEGF165 interaction. This concept is consistent with the observation that VEGF signaling is enhanced in β3 KO mice that lack integrin αvβ3 as a competitive modulator of VEGF165–KDR-driven signaling events. αvβ3 suppresses KDR D1 binding to the HBD. This is consistent with the observation that VEGF signaling is enhanced in β3 KO mice.

### 3.5 Full-length VEGF165 with a mutated HBD defective in αvβ3 and KDR D1 binding is defective in signaling functions while still binding KDR D2D3

**FIGURE 5:**
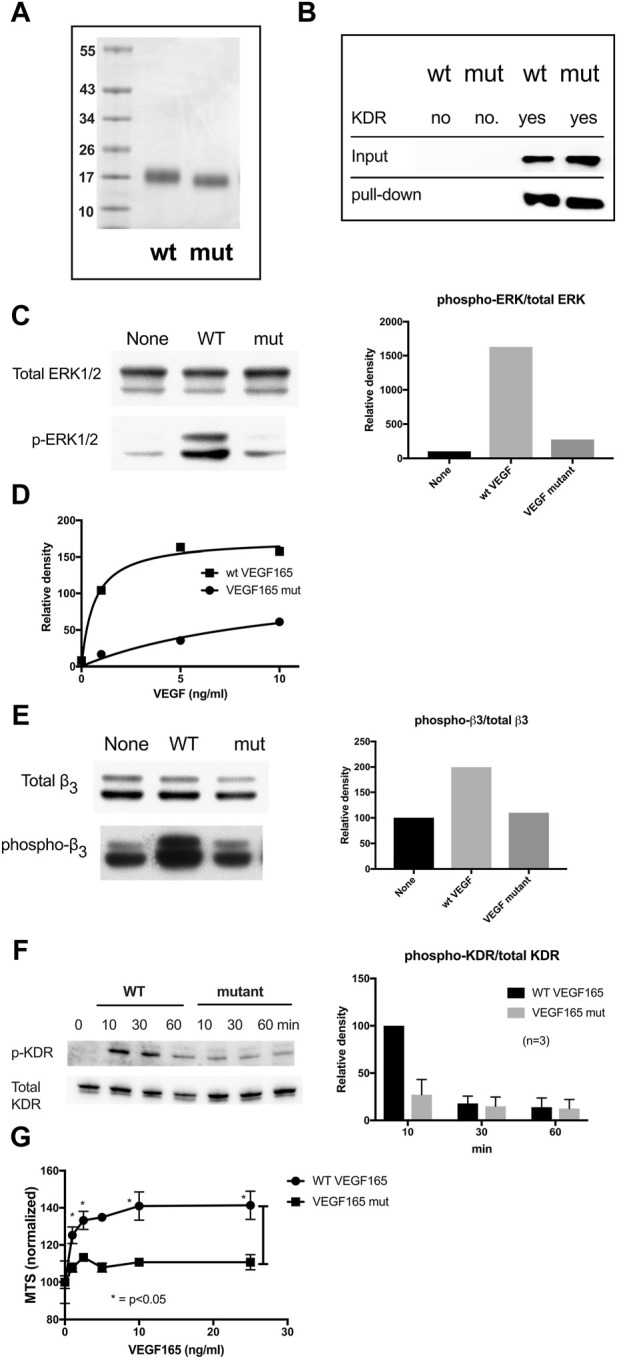
Partial characterization of the full-length VEGF165 mutant defective in integrin and KDR D1 binding **(A)**. SDS-PAGE analysis of wild-type and mutant VEGF165. In mutant VEGF165, all six amino acids within the HBD identified as required for integrin αvβ3 binding were changed to Ala (R123A/R124A/K125A/K140A/R145A/R149A). Molecular size values in kDa **(B)**. Pull-down of VEGF165 by KDR. His-tagged KDR was immobilized on Ni-NTR beads and incubated with full-length VEGF165 wild-type (WT) vs. VEGF165 mutant (mut) protein in binding buffer for 2 h at 4°C before elution and analysis of the retained protein by SDS-PAGE. Both wild-type and mutant VEGF165 bind to KDR. **(C)**. ERK1/2 activation in HUVECs by VEGF165. Starved HUVECs in M200 basal medium without a low serum growth supplement were treated with VEGF165 (10 ng/mL) for 10 min before lysis and Western blot analysis. Wild-type (WT) but not mutant (mut) VEGF165 activates ERK1/2 phosphorylation in human umbilical vein endothelial cells (HUVEC) **(D)**. Dose dependence of ERK1/2 activation in HUVECs by VEGF165. **(E)** Integrin αvβ3 phosphorylation in HUVEC in response to VEGF165. Starved HUVECs were treated with VEGF165 wild-type vs. mutant (10 ng/mL) before lysis and Western blot analysis of β3 integrin subunit phosphorylation. Wild-type (WT) but not mutant (mut) VEGF165 activates integrin αvβ3 phosphorylation in HUVECs. **(F)** KDR Y1175 phosphorylation in HUVEC in response to VEGF165. In HUVECs, wild-type (WT) but not mutant (mut) VEGF165 activates KDR Y1175 phosphorylation known to stimulate endothelial cell proliferation and migration. Starved HUVECs were treated for Western blot analysis as in panel **(E,G)**. VEGF165-induced proliferation of HUVEC. HUVECs were cultured overnight in M200 basal medium with 1% FBS and treated with WT or mutant VEGF165 for 4 days. Cell proliferation was measured using MTS assays.

Full-length VEGF165 protein with the combined HBD R123A/R124A/K125A/K140A/R145A/R149A mutations (referred to as VEGF165-HBD mutant) (Figure 5A) was synthesized in *E. coli* as an insoluble protein, purified under denaturation, re-folded, and further purified by FPLC gel filtration. The protein migrated as a single band with the expected size (Figure 5A). The protein bound to immobilized KDR in pull-down assays (Figure 5B), indicating that KDR binding specificity was retained by the VEGF165-HBD mutant protein. However, importantly, the VEGF165-HBD mutant lacked integrin binding αvβ3, as expected. Used as a soluble ligand for human endothelial cell (HUVEC) cultures, VEGF165-HBD mutant protein failed to induce ERK1/2 activation and integrin β3 phosphorylation in these cells (Figures 5C–E). Furthermore, VEGF165-HBD mutant protein also failed to activate KDR Y-1175 phosphorylation in HUVECs (Figure 5F) despite the ability of the mutant protein to bind to KDR (Figure 5B). Notably, VEGF165-HBD mutant protein failed to induce proliferation of HUVECs, which is consistent with the defective signaling functions of the mutant protein (Figure 5G). In the case of VEGF165 signaling, integrin αvβ3 appears to negatively regulate VEGF165 signaling by competing with the cognate receptor KDR for VEGF165 binding. Intriguingly, our results reveal a novel interaction of KDR with VEGF165 based on a competing mechanism mediated through KDR D1 binding to the ligand’s HBD, where the interaction overlaps with integrin αvβ3 binding. VEGF165 is probably the first example where integrin αvβ3 negatively regulates signaling induced by the growth factor interaction with a cognate cell-surface receptor.

## 4 Discussion

The present study identified amino acid residues within the VEGF165 HBD that are critical for integrin αvβ3 binding (Arg123, Arg124, Lys125, Lys140, Arg145, and Arg149) based on docking simulation and mutagenesis. Mutations R123A/R124A/K125A and K140A/R145A/R149A effectively reduced integrin binding. Combined mutations of all these amino acids (R123A/R124A/K125A/K140A/R145A/R149A) nearly completely suppressed the binding of αvβ3 to the VEGF165 HBD. Shedding new light on the mechanisms through which VEGF165 interacts with its cognate receptor KDR and exerts its mitogenicity, we discovered that the D1 domain of KDR binds to the VEGF165 HBD in addition to this ligand’s interaction with the KDR D2D3 domains, known to bind to the NTD of VEGF165. Consequently, our findings indicate that this complex interaction between KDR and VEGF165 is required for maximal induction of VEGF165-induced endothelial signaling responses. We discovered that KDR D1 bound to the HBD and the KDR D1-binding site and αvβ3-binding site overlap. The results from our study suggest that VEGF165 binds to KDR D1 via the HBD and to KDR D2D3 via the NTD and, as a result, induces the formation of a KDR D2D3–VEGF165-KDR D1 complex at the cell surface.

Full-length VEGF165 with the combined HBD mutations (R123A/R124A/K125A/K140A/R145A/R149A) did not bind to αvβ3 or KDR D1 but still bound to KDR D2D3. However, VEGF165 NTD binding to KDR D2D3 failed neither to induce EFK1/2 activation or phosphorylation of the integrin β3 subunit nor did this interaction result in KDR phosphorylation in HUVECs. Thus, we predict that the VEGF mutant may be a potential antagonist for VEGF signaling.

The role of integrin αvβ3 in VEGF165 signaling is still controversial since genetic deletion of β3 has been shown to unexpectedly enhance angiogenesis and tumorigenesis (Reynolds et al., 2002) and strengthen wound healing (Reynolds et al., 2005). Thus, it has been proposed that integrin αvβ3 negatively regulates VEGF signaling, although the mechanism of negative regulation had so far remained unclear. Our results provide a novel mechanism through which cell signaling in response to VEGF165 ligation to KDR is modulated by integrin αvβ3. Since we documented that KDR D1 can bind to the VEGF165 HBD and that the binding site for KDR D1 within the ligand’s HBD overlaps with the binding site of integrin αvβ3 within the VEGF165 HBD, αvβ3 most likely can act as a negative modulator of KDR-VEGF165-induced signaling. Our results support the concept that KDR D1 binding to the VEGF165 HBD is critical for VEGF165 signaling and that αvβ3 negatively regulates VEGF165 signaling by competing with KDR D1 for binding to the ligand’s HBD. Consistent with this notion, the KD of KDR D1 binding and that of αvβ3 binding to the VEGF165 HBD were comparable (approx. 10^–7^ M).

We previously reported that FGF1 and FGF2 directly bind to integrins and that these interactions lead to ternary complex formation between the integrin, the growth factor, and the cognate growth factor receptor (integrin–FGF–FGFR), which is required for their signaling functions (the ternary complex model) (Mori et al., 2008; Yamaji et al., 2010; Mori et al., 2013; Mori et al., 2017). This suggests that direct binding of integrins to FGF, which positively regulates FGF signaling, is required. Consistently, the mutant of FGF that abrogates integrin binding is functionally defective and suppresses signaling induced by WT FGF (dominant-negative antagonists) (Yamaji et al., 2010; Mori et al., 2013). These findings are consistent with the reports that antagonists to integrins, such as αvβ3, block angiogenesis induced by FGF2 (Brooks et al., 1994). Furthermore, we previously reported that integrins also directly bind to several growth factors other than FGF and positively regulate their signaling functions. Importantly, these growth factors include IGF-1 and -2, neuregulin-1, fractalkine, and CD40L, known as prominent regulators of tissue viability and metabolism, immune response, inflammation, and malignancy (Saegusa et al., 2009; Ieguchi et al., 2010; Fujita et al., 2012; Fujita et al., 2013; Cedano Prieto et al., 2017; Takada et al., 2019). We demonstrated that growth factor mutants defective in integrin binding (e.g., IGF1, IGF2, fractalkine, and CD40L) acted as dominant-negative antagonists (Fujita et al., 2012; Fujita et al., 2013; Cedano Prieto et al., 2017). Thus, it is likely that integrins positively regulate the signaling from these growth factors through direct binding to growth factors and ternary complex formation. In the case of VEGF165 signaling, integrin αvβ3 appears to negatively regulate VEGF165 signaling by competing with the cognate receptor KDR for VEGF165 binding. Intriguingly, our results reveal a novel interaction of KDR with VEGF165 based on a competing mechanism mediated through KDR D1 binding to the VEGF165 HBD, where the interaction overlaps with integrin αvβ3 binding. VEGF165 is probably the first example where integrin αvβ3 negatively regulates signaling induced by a growth factor interaction with a cognate cell-surface receptor.

## Data Availability

The original contributions presented in the study are included in the article/Supplementary Material, further inquiries can be directed to the corresponding author.
