# Supplementary material for: The heparin-binding domain of VEGF165 directly binds to integrin αvβ3 and VEGFR2/KDR D1: a potential mechanism of negative regulation of VEGF165 signaling by αvβ3
Source: Front Cell Dev Biol. 2024 May 9;12:1347616. doi: 10.3389/fcell.2024.1347616 (PMC11128890; doi:10.3389/fcell.2024.1347616)
Supplement: Supplementary file 1 [file DataSheet1.PDF]

a)

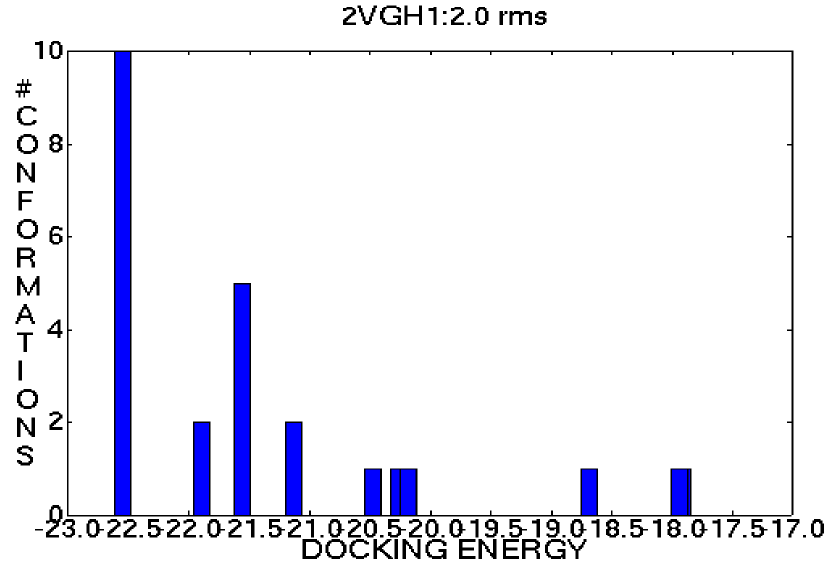
2VGH1:2.0 rms
CONFORMATIONS
0
2
4
6
8
10
-23.0
-22.5
-22.0
-21.5
-21.0
-20.5
-20.0
-19.5
-19.0
-18.5
-18.0
-17.5
-17.0
DOCKING ENERGY

b)

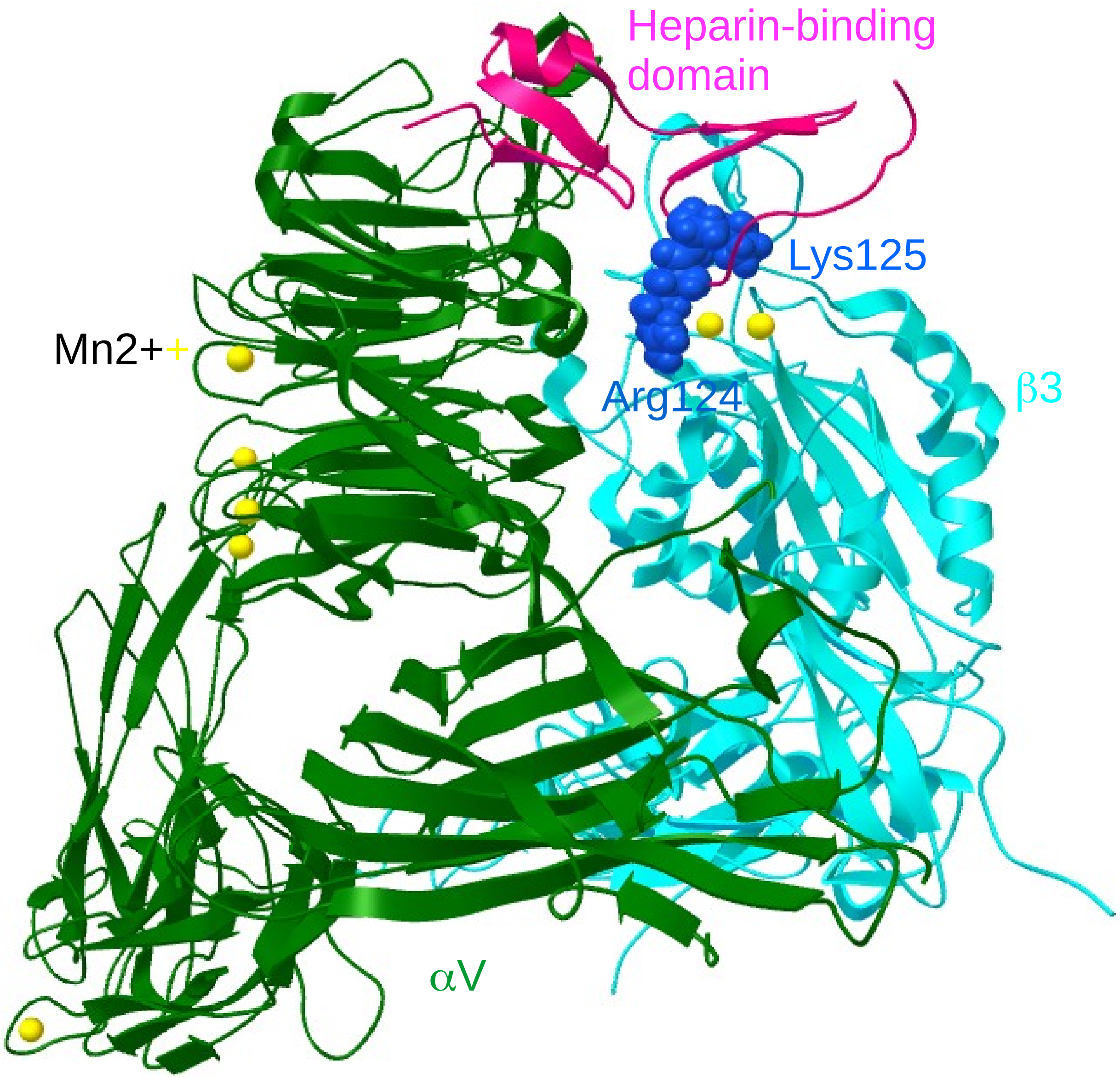
Heparin-binding domain
Lys125
Mn2++
Arg124
β3
αV

Supplemental Fig. S1

Docking simulation of interaction between the heparin-binding domain (HBD, 2VGH.pdb) and integrin αvβ3 (1L5G) with eight cations (Mn^{2+}) in the integrin molecule. (a) Clustering. The first cluster contains 10 poses (docking energy -25.5 Kcal/mol)(<2 rms) out of 25 poses. (b) Docking model of the pose in cluster 1. The HBD (magenta), Mn^{2+} (yellow). Integrin αv (green), and integrin β3 (light blue). Arg124 and Lys125 of the HBD are shown in blue. The pose of the first cluster is essentially identical with that of docking simulation (Fig. 2), in which all cations in the integrin molecule were removed.
